# Family Participation in the Nursing Care of the Hospitalized Patients

**DOI:** 10.5812/ircmj.12868

**Published:** 2014-01-05

**Authors:** Shahla Khosravan, Behnam Mazlom, Naiemeh Abdollahzade, Zeinab Jamali, Mohammad Reza Mansoorian

**Affiliations:** 1 Faculty of Nursing and Midwifery, Gonabad University of Medical Sciences, Gonabad, IR Iran; 2 Social Determents of Health Research Center, Gonabad University of Medical Sciences, Gonabad, IR Iran; 3 Department of Biostatics, Gonabad University of Medical Sciences, Gonabad, IR Iran; 4 Student Research Committee, Gonabad university of Medical Sciences, Gonabad, IR Iran

**Keywords:** Family Nursing, Hospitalization, Nursing Care

## Abstract

**Background:**

Few studies, especially in Iran, have assessed the status of family participation in the care of the hospitalized patients.

**Objectives:**

This study was conducted to assess why family members partake in caregiving of their patients in hospitals, the type of care that family provide, and the outcomes of the participation in the opinions of nurses and family members.

**Patients and Methods:**

In this comparative-descriptive study, data was collected by a two- version researcher-developed questionnaire, from 253 family members of patients by quota sampling method and 83 nurses by census sampling method from wards which had licensed for entering the families. Each questionnaire has three sections: the care needs of the patients which family participated to provide, the reasons to take part, and the outcomes of this collaborative care. The data was analyzed using descriptive statistics and also chi-squared test through SPSS software version 11.5.

**Results:**

The patients received more unskilled and non- professional nursing care from their family members. Most of the nurses and families believed that family participation is both voluntary and compulsory. The shortage of personnel in different categories of nursing and speeding up the patient-related affairs were the most important outcome of the participation, from the nurses’ viewpoint was speeding up the patient-related affairs and from the side of the family members, it was the patients’ feeling of satisfaction from the presence of one of their relatives beside them.

**Conclusions:**

Co understanding, skillfulness and competence of families and nurses in collaboration with each other were not good enough.Few studies, especially in Iran, have assessed the status of family participation in the care of the hospitalized patients.

## 1. Background

The main and final goal of nursing is providing the human health. Achieving this goal is possible through scientific care, and also suitable relation of nurses with patient and her/his family (1). It has been emphasized to facilitate the conditions of family’ participation and engagement in the care of hospitalized patients (2) due to the history of living the family members with the patient and their knowledge and skills in providing care for the patient (3). Providing this opportunity needs voluntary care of one of the family members and receiving help from personnel which is one of the needs of the family (2). In recent years, health care professionals have accepted appropriate treatment relation and interact between the family and the medical team at the hospital as one of the fundamental components of care (4) and the philosophy of family- centered care has been considered (5, 6).

While improving the quality of nursing care and more satisfaction of the patients and nurses, as the desired effects of family involvement in the nursing care of patients, have been reported in many studies (7-9), but it should be noted that neglecting the needs of the patients' families and imposing them more responsibilities than their capabilities have adverse effects such as feelings of inadequacy and inability (10). Few studies have assessed status of participation of the patients' family (11) and family and nurses view points in the role of families in caring of their relatives (12). In particular, there is not enough reliable evidence on the status of participation of the families in the care of their patients as one of the key indicators of family- centered care (13) in Iran.

## 2. Objectives

The main goal of this study was to assess the family participations status in the care of hospitalized patient from the nurses’ and the families’ points of view. In this regard, three objectives are considered to determine: The reason that a family participates in caring their patient in hospital, the type of the care and the outcomes of the participation.

## 3. Patients and Methods

A comparative- descriptive study was performed in two teaching hospitals, 22 Bahman and 15 Khordad related to Gonabad University of Medical Sciences, in 2011-2012 in Gonabad city, in Iran. The samples of this study were nurses who worked in the wards which had license for entering the patient’s family (internal, surgery, emergency, pediatrics, Obstetrics & Gynecology, and ENT), and families or relatives of patients who had been hospitalized in these wards. Nurses were selected by census sampling; inclusion criteria for nurses was having a bachelor's degree or higher and also having the experience of caring the patients with their family participation (No: 84). The patients’ family was selected by quota sampling method to reach to sample size (No: 253), through determining the share of each ward according to auditing calculation of occupied beds in the wards and according to the statistics of the number of patients hospitalized in the above mentioned sections at the first six months of 2011. Inclusion criteria for them was having experience of caring their patients at least one shift in one of the wards in two hospitals and aged at least 18 years who can read and write Persian or answer to the questionnaires orally.

The research tools were two researcher-made questionnaires for nurses and patient families. The questions were provided according to a primary interview with the families and the nurses working in the mentioned hospitals, job descriptions of the nursing personnel in Iran and also searching the references. Content and face validity of the questionnaire was further enhanced by submitting it to nursing research and education experts. The reliability of the questionnaires has been assessed by test- retest method and reliability coefficient of 0.7 was obtained for nurses’ version and 0.9 for patient families’ version. Each questionnaire consisted of demographics and three sections with 70 questions: the patients’ care needs for which family participated to provide, the reasons of participation, and the outcomes of collaborative care. The first section of nursing care included 38 questions in five areas: care (11 question), treatment (9 question), supportive – protective (10 question), coordinative (4 question), teaching (4 question); each second and third sections included 16 questions.

This study was approved by the Ethics committee of Gonabad University of Medical Sciences (Code No: 90/8, Date: 15.9.2011). The questionnaires were completed and collected after receiving oral satisfaction with full awareness about the goals of the study No individual identifiable information was included on either the questionnaires or demographics sheet. To consider Patient Privacy, data collections from the families were done at the time of discharge of their patient from hospital. Statistical analysis was carried out using SPSS 11.5 software and the results analyzed using descriptive statistics of frequency, relative frequency, mean and also chi-squared test P < 0.05 was considered statistically significant.

## 4. Results

The demographic results showed that from 84 nurses, 14.2% were male and 85.7% female with 1-20 years’ experience in nursing with bachelor degree. 16 of them worked in internal ward, 19 in surgery, 23 in emergency, 12 in pediatrics, 11 in Obstetrics & Gynecology, and 3 in ENT ward. From 253 members of the families, 33.6% were male and 66.4% female; more details was shown in Table 1. 

**Table 1.** Demographic Variables Related to Family Members of Patients

Variables	No.	%
**Gender**		
Female	168	66.4
Male	85	33.6
Total	253	100
**Age (Year)**		
20- 29	137	54.1
30-39	92	36.4
40 and more	24	9.5
Total	253	100
**Education**		
Illiterate	69	27.3
Diploma and less	154	60.8
Higher Education	30	11.9
Total	253	100
**Residence**		
Rural	143	56.5
Urban	110	43.5
Total	253	100
**Ward**		
Surgery	38	15
Internal	64	25.2
Urgency	65	25.7
Obstetrics and Gynecology	35	13.8
ENT	23	9
Pediatric	28	11.3
Total	253	100
**Hospital**		
22 Bahman	215	84.9
15 Khordad	38	15.1
Total	253	100

The familys’ participation for performing patients’ care affairs was more in night shifts comparing to the morning shifts. Only 3.6% of nurses and 7.6% of the families believed that performing the affairs by the families is compulsory, while 44% of the nurses and 45.5% of the families believed that performing the affairs by the families is completely voluntary. Other nurses and family believed that performing the affairs by the family is both voluntary and compulsory. In relation to the first objective, determining the type of participated nursing duties, the results showed that according to the viewpoint of the families, they participated in performing care (37.62), coordinative (30.04), teaching affairs (28.06), supportive – protective (26.40), and treatment (18.93), while in the viewpoint of the nurses, the families’ participated duties were care (43.06), supportive – protective (30.06), treatment (20.04), teaching (14.26) and coordinative affairs (12.20), respectively. Therefore, from the viewpoints of the nurses and families, the care affair was the most common family’s participated duties and among these affairs, moving and feeding the patient (92.7) were as in priority; others were shown in Table 2. Results related to the causes of participation of families in patients’ care as the second objective and determining the outcomes of participation from the viewpoint of the nurses and the families are shown in Tables 3 and 4 respectively. 

**Table 2.** Nurses and Families Perspective About the Type of Participated Care Affairs

Care Affairs	Patient’s Family Assigned		Nurse Assigned	
	No.	%	No.	%
**Taking the samples to laboratory**	155	61.3	45	53.6
**Emptying the urine bag**	91	36	60	71.6
**Arising the patient and feeding patient**	205	81	78	92.9
**Changing the cloth**	171	67.6	42	50
**Changing the bed sheet**	41	16.2	3	3.6
**Active and inactive exercises**	87	34.4	22	26.2
**Placing the temperature**	51	20.4	0	0
**Placing and removing the basin for defecation**	120	47.4	69	82.2
**Changing children diaper and cleaning them**	53	20.9	47	56
**Patient’s respiratory physiotherapy**	35	13.8	10	11.9

**Table 3.** Comparison of Nurses and Families Perspective About the Causes of Assigning

Groups	Agreed Nurses, n = 84		Agreed Family, n = 253		P value	Odds Ratio	Confidence Interval	
	No.	%	No.	%			Low	Upper
**Not having enough time**	43	51	53	20	0.999	0.253	0.150	0.427
**Speeding up the patient-related affairs**	44	52	147	58	0.359	1.261	0.768	2.070
**Ward being busy**	58	69	92	39.4	0.999	0.256	0.151	0.435
**Shortage of personnel in different categories of nursing**	58	72	77	30	0.999	0.165	0.095	0.286
**Many specialized tasks causes assigning non-specialized tasks**	30	35.7	28	11.1	0.999	0.224	0.124	0.406
**Nurse fatigue**	16	19	43	17	0.667	0.870	0.461	1.643
**Too much attention of the nurses to the accounting tasks such as writing files**	22	26.2	64	25.3	0.871	0.954	0.543	1.676
**Neglect and carelessness of the personnel**	7	8.3	46	18.2	0.032	2.444	1.058	5.646
**Routinely performing the patient’s tasks by the family**	28	3/33	50	8/19	0.011	0.493	0.284	0.583
**Not informed about duties**	3	3.6	51	20	0.999	6.817	2.068	22.465
**Working in several consecutive shifts**	17	20.2	47	18.6	0.737	0.899	0.484	1.671
**Unsuitable wage rather than high volume of the tasks**	10	11.9	23	9.1	0.452	0.740	0.337	1.626
**keeping the patient’s privacy and religious problems**	26	31	79	31.2	0.963	1.013	0.594	1.727
**Not coordinating of the patient with hospital personnel**	10	11.9	50	19.8	0.103	1.823	0.879	3.779
**For more participation as the nearest person beside patient**	34	40.5	128	50.6	0.108	1.506	0.913	2.484
**Family’s high level of education**	6	7.1	21	8.3	0.735	1.177	0.485	3.021

**Table 4.** Comparison of Nurses and Families Perspective About the Outcomes of Assigning the Affairs to Patient’s Family

Outcomes	Agreed Nurses, n = 84		Agreed Family, n = 253		P value	Odds Ratio	Confidence Interval	
	No.	%	No.	%			Low	Upper
**Not correctly performing the assigned duty**	34	26.9	68	26.9	0.019	0.541	0.322	0.906
**Duplication**	26	22.2	56	22.2	0.107	0.637	0.368	1.104
**Decreasing the nurse value**	16	13.8	35	13.8	0.248	0.682	0.365	2.094
**Family no satisfaction from the patient hospitalization in hospital**	15	19.4	49	19.4	0.760	1.105	0.583	2.094
**Damage to patient’s health **	11	29.6	75	29.6	0.003	2.796	1.404	5.569
**Request for discharge before the determined time**	8	19	48	19	0.044	2.224	1.006	4.918
**Speeding up the patient-related affairs**	53	43.9	111	43.9	0.002	0.457	0.275	0.760
**Respect for patient privacy and religious issues**	31	36.4	92	36.4	0.929	0.977	0.580	1.630
**The patient’s feeling of satisfaction from the presence of family**	47	69.6	176	69.6	0.090	1.554	0.932	2.592
**Participation and interaction between nurse and patient’s family**	43	42.3	97	42.3	0.155	0.699	0.426	1.147
**The feeling of being useful as the patient’s family**	24	49.4	125	49.4	0.001	2.441	2.432	4.163
**Increasing the patient’s costs**	1	1.2	33	13	0.002	12.450	1.676	92.491
**Physical and verbal confrontation with nurse**	9	10.7	28	11.1	0.929	1.037	0.468	2.297
**Intervention in treatment**	19	22.6	39	15.4	0.130	0.623	0.337	1.153
**Causing problem for patient’s health**	7	8.3	65	25.7	0.001	3.803	1.669	8.666
**Increasing the social respect for nurse personnel**	7	8.3	68	26.9	0.999	4.043	1.777	9.200

## 5. Discussion

The results of this study showed that family often voluntary participated in the caring for relatives, especially in the primary care (Table 1). The results of other researches also suggested that family cares perceived themselves to have a greater role in caring for relatives were mostly satisfied with their role (11). According to Aiken et al. (14), and also based on the job description of nurses (15), most of the assigned primary care affairs are the duties of assisted nurses. Based on the results of this study, among other tasks, the specialized treatment affairs such as injections and dressing which are the main tasks of educated nurses in Iran are the least affairs assigned to the patient’s family. The results of other study in other countries also showed that primary care affairs were performed by families and specialized affairs were carried out by nurses (11, 12). Due to this situation patients frequently complained about the lack of care needs by nurses and used the support of one of their family members for doing these care affairs (11).

The results of this study also showed that doing the affairs for the patient were in the rate of mainly involuntary to voluntary condition. While, other results showed that despite most of the families of hospitalized children (mothers) wanted the desired care by the nurses for their child, but this was not performed and finally doing was assigned to the mothers (16). The other study suggests that families' tendency to participate in patient care is very important morally and the families don’t have any responsibility for the care of patients legally; therefore, they should not be asked to do more than what they want or they can do (17); also desire and tendency of the family for involvement in patient’s care does not mean that they have enough ability to provide adequate care or willingness to perform the imposed responsibilities, rather they want to do the care affairs voluntarily with help of the personnel (18, 19). In this study, both nurses and families believed that the most common cause of assigning some of the cares to the families is shortage of personnel in different categories of nursing and the families accept these care affairs either mandatory or optional to perform their patient’s care affairs quickly. The result of other study showed that this shortage includes different categories of professional nurse and assisted nurse and the shortage of nurse group leads to using of the families for performing nursing care (20-22). Also the results showed that the families and nurses reported that consecutive shifts of the personnel are the cause of assigning some of the nursing care to the families. Other study also demonstrated mandatory and voluntary overtime (more than 40 hours in week) could negatively impact nurse and patient safety, and significantly related with adverse events and errors, including needlestick injuries, work-related injuries, patient falls with injury, nosocomial infections, and medication errors (23).

Our study showed that the most common cause of assigning some of the cares being lack of time related to focusing the nurse on tasks such as documentation. Other study also suggested that, nurses are faced with multiple tasks and much of the theirs’ time has been spent on the non-specialized tasks; they are engaged in tasks that are more similar to secretary duties and therefore, the main task of professional nurse and meaningful relationships with patients and their families has not been made due to the shortage of time (24). Maintaining the privacy of patient and providing care by the nurses of the same gender, related to Islamic religious culture in Iran, is another factor that suggested the necessity of participation of family to provide care when there is no such nurse. The theme of noticing to culture (25) Islamic values in providing the nursing care based on professional ethics have suggested in other studies (26, 27).

 The results of this study showed that participation in providing care has positive consequences such as accelerating the performance of the affairs, and also the negative consequences such as not correctly performing of assigning task, damage to patient’s health and their relatives. According to other study, although providing care by the family can make feeling of security and comfort for the patient, but in some cases, due to lack of sufficient information of the family, it leads to the patient’s worry some and a sense of insecurity (28) and also families are faced with a constant tension due to forced and imposed care of their patients (16, 18, 19). Increasing the patient’s costs and request for discharge before the determined time was another consequences of this research from viewpoint of families. According to other researchers increasing hospital costs associated with the presence of family provides the background for dissatisfaction and complaint (18, 29), and in some cases, leads to verbal and physical violence against nurses by patients and their family (30-33)

In this study, causing health problems for the families was one of the outcomes of assigning the affairs. According to other studies neglecting the needs of the patients' families due to poor physical environment and lack of hospital facilities to meet the minimum basic needs of human health (34) have negative effect on the family’s health and imposing over responsibility to them have adverse effects such as feelings of inadequacy, inability (9), psychological burden (29), anxiety, depression (35), tiredness and feeling of being alone in their patient care responsibilities (4). Although this study tried to present the status of participation of the families in the care of their patients, but the data was collected by a self- report questionnaire with a convenience sampling strategy. We recommended data gathering method improve in following studies.

Interaction and relation of the families with the hospital treatment team is one of the essential components of care; but this need was not properly met; effective presence of the nurse can balance the role of the family beside the patient. In this regard, training the personnel for better and more understanding of the families and involving them in the care based on their abilities according to the philosophy of family- centered care has been suggested.
